# Supplementary material for: Rural-urban outcome differences associated with COVID-19 hospitalizations in North Carolina
Source: PLoS One. 2022 Aug 17;17(8):e0271755. doi: 10.1371/journal.pone.0271755 (PMC9384999; doi:10.1371/journal.pone.0271755)
Supplement: Attachment — Submitted filename: Reviewer attachments.docx [file pone.0271755.s001.docx]

Anna Volynkina,

Head of Laboratory of Viral Infections

Diagnostic,

Stavropol Research Antiplague Institute,

355035, Stavropol, Russian Federation

27.03.2022

Dear Dr. Robert Jeenchen Chen,

The paper by Sheri Denslow et al. is devoted to the analysis of differences in the outcome of the disease in patients with Сovid-19 from rural and urban areas in in North Carolina. As a result of the study, the conclusion that rural North Carolina residents hospitalized for COVID-19 had a higher probability of dying, which is associated with higher rates of comorbidities in rural patients.

I think that the manuscript can be accepted for publication after some changes have been made:

1. It is necessary to analyze the relationship between the COVID-19 outcome and the timeliness of rural and urban patients hospitalization after the onset of symptoms (patients were hospitalized within a day after the disease, or on the third, fifth, tenth day). It is possible that untimely hospitalization of rural patients is associated with fatal outcomes of Covid-19.

2. It is necessary to add in the Discussion whether the conclusions obtained as a result of the work will change if we analyze the data on patients in 2021-2022. During this period (2021-2022), different genetic variants of SARS-CoV-2 virus circulated in the World, affecting the epidemiological and clinical features of the COVID-19. For example, the Delta and Omicron variants infected younger people that other variants. The Delta variant caused a more severe course of the disease that other variants.

Kind regards,

Anna Volynkina
