# Supplementary material for: Rural-urban outcome differences associated with COVID-19 hospitalizations in North Carolina
Source: PLoS One. 2022 Aug 17;17(8):e0271755. doi: 10.1371/journal.pone.0271755 (PMC9384999; doi:10.1371/journal.pone.0271755)
Supplement: Attachment — Submitted filename: Response to Reviewers.docx [file pone.0271755.s002.docx]

May 2, 2022

Dear Robert Jeenchen Chen, MD, MPH, Academic Editor, PLOS ONE

Thank you for the opportunity to revise and resubmit our original research article (PONE-D-21-29033) entitled, **Rural-urban outcome differences associated with COVID-19 hospitalizations in North Carolina.** We appreciate the editor’s and reviewers’ valuable input. Our responses (in blue text) to the each of the reviewers’ points is below the reviewer’s statement (indented, black text). All suggested changes have been made in the revised manuscript (highlighted in yellow).

Please let me know if any further information is needed.

Sincerely,

Jason Wingert, PhD, MPT

Professor of Health and Wellness Promotion

University of North Carolina Asheville

When submitting your revision, we need you to address these additional requirements.

1. Please ensure that your manuscript meets PLOS ONE's style requirements, including those for file naming.

We have ensured that our manuscript meets these requirements.

2. We note that the grant information you provided in the ‘Funding Information’ and ‘Financial Disclosure’ sections do not match. When you resubmit, please ensure that you provide the correct grant numbers for the awards you received for your study in the ‘Funding Information’ section.

Upon resubmission, we will ensure that all information provided in the ‘Funding Information’ and ‘Financial Disclosure’ sections do match.

3. Please state what role the funders took in the study.  If the funders had no role, please state: "The funders had no role in study design, data collection and analysis, decision to publish, or preparation of the manuscript."

The above statement now appears in our cover letter, as requested.

4. In your Data Availability statement, you have not specified where the minimal data set underlying the results described in your manuscript can be found. PLOS defines a study's minimal data set as the underlying data used to reach the conclusions drawn in the manuscript and any additional data required to replicate the reported study findings in their entirety. All PLOS journals require that the minimal data set be made fully available. For more information about our data policy, please see <http://journals.plos.org/plosone/s/data-availability>.

Upon re-submitting your revised manuscript, please upload your study’s minimal underlying data set as either Supporting Information files or to a stable, public repository and include the relevant URLs, DOIs, or accession numbers within your revised cover letter. For a list of acceptable repositories, please see <http://journals.plos.org/plosone/s/data-availability#loc-recommended-repositories>. Any potentially identifying patient information must be fully anonymized.

Important: If there are ethical or legal restrictions to sharing your data publicly, please explain these restrictions in detail. Please see our guidelines for more information on what we consider unacceptable restrictions to publicly sharing data: <http://journals.plos.org/plosone/s/data-availability#loc-unacceptable-data-access-restrictions>. Note that it is not acceptable for the authors to be the sole named individuals responsible for ensuring data access.

We will update your Data Availability statement to reflect the information you provide in your cover letter.

A letter detailing our legal restrictions on publicly sharing these data will be sent to the editor upon resubmission of our revised manuscript.

5. We note that you have indicated that data from this study are available upon request. PLOS only allows data to be available upon request if there are legal or ethical restrictions on sharing data publicly. For more information on unacceptable data access restrictions, please see <http://journals.plos.org/plosone/s/data-availability#loc-unacceptable-data-access-restrictions>.

In your revised cover letter, please address the following prompts:

a) If there are ethical or legal restrictions on sharing a de-identified data set, please explain them in detail (e.g., data contain potentially sensitive information, data are owned by a third-party organization, etc.) and who has imposed them (e.g., an ethics committee). Please also provide contact information for a data access committee, ethics committee, or other institutional body to which data requests may be sent.

b) If there are no restrictions, please upload the minimal anonymized data set necessary to replicate your study findings as either Supporting Information files or to a stable, public repository and provide us with the relevant URLs, DOIs, or accession numbers. For a list of acceptable repositories, please see <http://journals.plos.org/plosone/s/data-availability#loc-recommended-repositories>.

We will update your Data Availability statement on your behalf to reflect the information you provide.

A letter detailing our legal restrictions on publicly sharing these data will be sent to the editor upon resubmission of our revised manuscript. These restrictions will also be described in our cover letter, following the prompt 5a above.

6. Please include your full ethics statement in the ‘Methods’ section of your manuscript file. In your statement, please include the full name of the IRB or ethics committee who approved or waived your study, as well as whether or not you obtained informed written or verbal consent. If consent was waived for your study, please include this information in your statement as well.

The Methods section contains the full names of all reviewing IRBs and a statement clarifying that consent was waived for this study on Page 5-6, Line 147-152.

7. We note that [Figure 1] in your submission contain [map/satellite] images which may be copyrighted. All PLOS content is published under the Creative Commons Attribution License (CC BY 4.0), which means that the manuscript, images, and Supporting Information files will be freely available online, and any third party is permitted to access, download, copy, distribute, and use these materials in any way, even commercially, with proper attribution. For these reasons, we cannot publish previously copyrighted maps or satellite images created using proprietary data, such as Google software (Google Maps, Street View, and Earth). For more information, see our copyright guidelines: <http://journals.plos.org/plosone/s/licenses-and-copyright>.

We require you to either (1) present written permission from the copyright holder to publish these figures specifically under the CC BY 4.0 license, or (2) remove the figures from your submission:

Upon submission of the revised manuscript, we will present written permission from the copyright holder to publish these figures specifically under the CC BY 4.0 license, uploaded as an “Other” file with our submission.

In the figure caption of the copyrighted figure, please include the following text: “Reprinted from [ref] under a CC BY license, with permission from [name of publisher], original copyright [original copyright year].”

We have added this caption to Figure 1.

Reviewers' comments:

Reviewer's Responses to Questions

**Comments to the Author**

1. Is the manuscript technically sound, and do the data support the conclusions?

The manuscript must describe a technically sound piece of scientific research with data that supports the conclusions. Experiments must have been conducted rigorously, with appropriate controls, replication, and sample sizes. The conclusions must be drawn appropriately based on the data presented.

Reviewer #1: Partly

Reviewer #2: Yes

2. Has the statistical analysis been performed appropriately and rigorously?

Reviewer #1: Yes

Reviewer #2: Yes

3. Have the authors made all data underlying the findings in their manuscript fully available?

The [PLOS Data policy](http://www.plosone.org/static/policies.action#sharing) requires authors to make all data underlying the findings described in their manuscript fully available without restriction, with rare exception (please refer to the Data Availability Statement in the manuscript PDF file). The data should be provided as part of the manuscript or its supporting information, or deposited to a public repository. For example, in addition to summary statistics, the data points behind means, medians and variance measures should be available. If there are restrictions on publicly sharing data—e.g. participant privacy or use of data from a third party—those must be specified.

Reviewer #1: Yes

Reviewer #2: No

4. Is the manuscript presented in an intelligible fashion and written in standard English?

PLOS ONE does not copyedit accepted manuscripts, so the language in submitted articles must be clear, correct, and unambiguous. Any typographical or grammatical errors should be corrected at revision, so please note any specific errors here.

Reviewer #1: Yes

Reviewer #2: Yes

5. Review Comments to the Author

Please use the space provided to explain your answers to the questions above. You may also include additional comments for the author, including concerns about dual publication, research ethics, or publication ethics. (Please upload your review as an attachment if it exceeds 20,000 characters)

Reviewer #1: This is a useful descriptive study that is well presented and I have no concerns about the analysis. The concern about inequalities between urban and rural patients is important to examine, particularly in the light of disparities in funding the authors describe.

The main finding is that rural patients are sicker on admission than the urban patients, and adjusting for this moves the association with mortality closer to the null and within the range of being explained by unmeasured confounding. This suggests the key drivers are related to pre existing pre admission factors rather than what happens in hospital. The authors acknowledge this but that their study being hospital based is not able to examine these factors in detail.

However, the major concern is whether there is selection bias with less sick rural patients being less likely to be admitted than less sick urban patients. This is possible because the study is based in tertiary care rather than population based. Therefore, it is important to consider if there could have been any selection bias between rural and urban patients regarding who gets admitted to one of these large academic health systems. Is it possible to have missed less sick rural patients who might have been admitted to local smaller rural hospitals that were not part of the big academic health systems? Or for milder rural patients to have a greater tendency to be managed at home? In which case there would be a trend to only the sicker rural patients being admitted or transferred to one of the 3 large academic health systems. Therefore, there would be selection bias towards sicker rural patients compared to urban patients in the study population.

We appreciate this comment and our team had similar discussions throughout. However, using limited data sets that were provisioned from existing clinical data warehouses, we cannot provide additional variables that may help to disentangle these many factors that are likely confounders in our analysis. We acknowledge this in our initial manuscript’s limitations section text.

Which patients would not be eligible for admission to one of these health care systems from the rural and urban populations and would therefore be excluded from the study? If there is a disparity in this related to the rural / urban because of the differences in health insurance then again there could be selection bias towards sicker patients from a rural area because less sick rural patients would not be admitted to these hospitals.

Would there be a difference in decision making for escalation to higher levels care such as ICU, as opposed to palliative hospice care, that would be dependent on the different insurance levels?

Again, with limited data sets from existing clinical data, such important questions cannot be answered, but indeed a mixed methods approach could shed light on these critical questions.

Reviewer #2: The article is devoted to the analysis of differences in the outcome of the disease in patients with Сovid-19 from rural and urban areas in in North Carolina. As a result of the study, the conclusion that rural North Carolina residents hospitalized for COVID-19 had a higher probability of dying, which is associated with higher rates of comorbidities in rural patients.

I think that the manuscript can be accepted for publication after some changes have been made:

1. It is necessary to analyze the relationship between the COVID-19 outcome and the timeliness of rural and urban patients hospitalization after the onset of symptoms (patients were hospitalized within a day after the disease, or on the third, fifth, tenth day). It is possible that untimely hospitalization of rural patients is associated with fatal outcomes of Covid-19.

We fully appreciate this comment and agree that it would be ideal to understand the time to event from symptom onset to hospitalization, however, symptom onset is not a variable that is available as a discreet field in our health system data, thus we are not able to provide this data. We have included a statement describing this in the limitations section (Page 18, lines 358-361).

2. It is necessary to add in the Discussion whether the conclusions obtained as a result of the work will change if we analyze the data on patients in 2021-2022. During this period (2021-2022), different genetic variants of SARS-CoV-2 virus circulated in the World, affecting the epidemiological and clinical features of the COVID-19. For example, the Delta and Omicron variants infected younger people that other variants. The Delta variant caused a more severe course of the disease that other variants.

Response to reviewer comment #2:

We appreciate this suggestion and indeed the variants have changed since our data collection completed. We have added the following sentence to the Discussion section on Page 18, line 355-358:

Additionally, the effects of subsequent genetic variants of the SARS-CoV-2 virus, including the Delta and Omicron variants of 2021 and 2022, on hospitalizations and deaths could not be determined with data from this time period. Differences in outcomes between rural and urban patients resulting from later variants should be investigated in subsequent research.

6. PLOS authors have the option to publish the peer review history of their article ([what does this mean?](https://journals.plos.org/plosone/s/editorial-and-peer-review-process#loc-peer-review-history)). If published, this will include your full peer review and any attached files.


If you choose “no”, your identity will remain anonymous but your review may still be made public.


**Do you want your identity to be public for this peer review?** For information about this choice, including consent withdrawal, please see our [Privacy Policy](https://www.plos.org/privacy-policy).

Reviewer #1: No

Reviewer #2: No

[NOTE: If reviewer comments were submitted as an attachment file, they will be attached to this email and accessible via the submission site. Please log into your account, locate the manuscript record, and check for the action link "View Attachments". If this link does not appear, there are no attachment files.]

While revising your submission, please upload your figure files to the Preflight Analysis and Conversion Engine (PACE) digital diagnostic tool, <https://pacev2.apexcovantage.com/>. PACE helps ensure that figures meet PLOS requirements. To use PACE, you must first register as a user. Registration is free. Then, login and navigate to the UPLOAD tab, where you will find detailed instructions on how to use the tool. If you encounter any issues or have any questions when using PACE, please email PLOS at [figures@plos.org](mailto:figures@plos.org). Please note that Supporting Information files do not need this step.

 Additional Reviewer:

Anna Volynkina,

Head of Laboratory of Viral Infections

Diagnostic,

Stavropol Research Antiplague Institute,

355035, Stavropol, Russian Federation

27.03.2022

Dear Dr. Robert Jeenchen Chen,

The paper by Sheri Denslow et al. is devoted to the analysis of differences in the outcome of the disease in patients with Сovid-19 from rural and urban areas in in North Carolina. As a result of the study, the conclusion that rural North Carolina residents hospitalized for COVID-19 had a higher probability of dying, which is associated with higher rates of comorbidities in rural patients.

I think that the manuscript can be accepted for publication after some changes have been made:

1. It is necessary to analyze the relationship between the COVID-19 outcome and the timeliness of rural and urban patients hospitalization after the onset of symptoms (patients were hospitalized within a day after the disease, or on the third, fifth, tenth day). It is possible that untimely hospitalization of rural patients is associated with fatal outcomes of Covid-19.

We fully appreciate this comment and agree that it would be ideal to understand the time to event from symptom onset to hospitalization, however, symptom onset is not a variable that is available as a discreet field in our health system data, thus we are not able to provide this data. We have included a statement describing this limitation on Page 18, line 358-361.

2. It is necessary to add in the Discussion whether the conclusions obtained as a result of the work will change if we analyze the data on patients in 2021-2022. During this period (2021-2022), different genetic variants of SARS-CoV-2 virus circulated in the World, affecting the epidemiological and clinical features of the COVID-19. For example, the Delta and Omicron variants infected younger people that other variants. The Delta variant caused a more severe course of the disease that other variants.

Response to reviewer comment #2:

We appreciate this suggestion and indeed the variants have changed since our data collection completed. We have added a sentence describing this limitation to our analysis on Page 18, line 355-358.

Kind regards,

Anna Volynkina
