# Supplementary material for: Rural-urban outcome differences associated with COVID-19 hospitalizations in North Carolina
Source: PLoS One. 2022 Aug 17;17(8):e0271755. doi: 10.1371/journal.pone.0271755 (PMC9384999; doi:10.1371/journal.pone.0271755)
Supplement: Attachment — Submitted filename: Response to Reviewers 6.20.2022 2pm.docx [file pone.0271755.s003.docx]

Dear Robert Jeenchen Chen, MD, MPH, Academic Editor, PLOS ONE

Thank you for the review of our original research article (PONE-D-21-29033) entitled, **Rural-urban outcome differences associated with COVID-19 hospitalizations in North Carolina.** We appreciate the editor’s and reviewers’ continued input. Our response (in blue text) to the reviewer’s additional comment is below the reviewer’s statement (black text). All manuscript revisions are shown in the revised manuscript (highlighted in yellow).

Please let me know if further information is needed.

Sincerely,

Jason Wingert, PhD

University of North Carolina Asheville

Reviewers' comments:

Reviewer #3: This revised article, entitled “Rural-urban outcome differences associated with COVID-19 hospitalizations in North Carolina”, analyzed the differences in the
outcome of the disease in patients with Сovid-19 from rural and urban areas in in North Carolina. And the authors concluded that rural North Carolina residents
hospitalized for COVID-19 had a higher probability of mortality/hospice discharge, after adjustment of age, sex, race/ethnicity, payer, disease comorbidities, presenting oxygen levels and cytokine levels.

I’ve read both original and revised editions, and the authors made a very persuasive explanations and adjustment according to the previous reviewers’ comments. It will be better if it’s added a p value in tables, and clarify the relative ratio using crude model or adjusted model in figures.

Thank you for pointing out that we should specify that Figure 2 is showing unadjusted values. We have modified the Figure heading to state that this is reporting an unadjusted incidence.

For this primarily descriptive analysis, we chose to emphasize estimation over testing through reporting point estimates and confidence intervals and not reporting p-values. Please see articles by Amrhein, Greenland and McShane (Scientists rise up against statistical significance. Nature, 2019) and Ranstam (Why the p-value culture is bad and confidence intervals a better alternative. Osteoarthritis and Cartilage, 2012) for additional support of our decision.
